# Activation of Penile Proadipogenic Peroxisome Proliferator-Activated Receptor *γ* with an Estrogen: Interaction with Estrogen Receptor Alpha during Postnatal Development

**DOI:** 10.1155/2008/651419

**Published:** 2008-08-21

**Authors:** Mahmoud M. Mansour, Hari O. Goyal, Tim D. Braden, John C. Dennis, Dean D. Schwartz, Robert L. Judd, Frank F. Bartol, Elaine S. Coleman, Edward E. Morrison

**Affiliations:** ^1^Departments of Anatomy, Physiology, and Pharmacology, College of Veterinary Medicine, Auburn University, Auburn, AL 36849, USA; ^2^Department of Biomedical Sciences, Tuskegee University, Tuskegee, AL 36088, USA; ^3^Cellular and Molecular Biosciences Program, Department of Animal Sciences, Auburn University, Auburn, AL 36849, USA

## Abstract

Exposure to the estrogen receptor alpha (ER*α*) ligand diethylstilbesterol (DES) between neonatal days 2 to 12 induces penile adipogenesis and adult infertility in rats. The objective of this study was to investigate the in vivo interaction between DES-activated ER*α* and the proadipogenic transcription factor peroxisome proliferator-activated receptor gamma (PPAR*γ*). Transcripts for PPARs *α*, *β*, and *γ* and *γ*1a splice variant were detected in Sprague-Dawley normal rat penis with PPAR*γ* predominating. In addition, PPAR*γ*1b and PPAR*γ*2 were newly induced by DES. The PPAR*γ* transcripts were significantly upregulated with DES and reduced by antiestrogen ICI 182, 780. At the cellular level, PPAR*γ* protein was detected in urethral transitional epithelium and stromal, endothelial, neuronal, and smooth muscular cells. Treatment with DES activated ER*α* and induced adipocyte differentiation in corpus cavernosum penis. Those adipocytes exhibited strong nuclear PPAR*γ* expression. These results suggest a biological overlap between PPAR*γ* and ER*α* and highlight a mechanism for endocrine disruption.

## 1. INTRODUCTION

Endocrine disruption, originally limited to steroid receptor signaling, now extends to include other members of the 48 reported nuclear receptor superfamily [1]. Both peroxisome proliferator-activated receptor gamma (PPAR*γ*) and estrogen receptor alpha (ER*α*) are targets for endocrine disrupting chemicals [2–4]. Recently, Goyal et al. showed that neonatal exposure of rats to the estrogenic endocrine disruptor diethylstilbestrol (DES) induced adipogenesis in penile corpus cavernosum by activation of ER*α* [5–8]. In this model of DES-ER*α* activation, DES exposure at a dose of 0.1 to 0.12 mg/kg bw/day, on alternate days, from postnatal days 2 to12, resulted in infertility in 100% of the treated male rats. Loss of fertility was associated with abnormal accumulation of fat cells in the corpus cavernosum penis, and the associated loss of cavernous spaces apparent as early as postnatal day 18 (reviewed in [9]). It remains unknown, however, whether this penile ER*α*-induced adipogenesis is mediated by activation of a constitutively expressed or DES-induced PPAR*γ*.

Both ER*α* and PPAR*γ* pathways are implicated in fat regulation. First, recent findings suggest that PPAR*γ* and ER*α* pathways involve shared coactivators that promote differentiation of preadipocytes into mature fat cells. For example, constitutive coactivator of PPAR*γ* (CCPG) is described as a *bona fide* coactivator that cross reacts with ER*α* independent of its ligand and contains four *LXXLL* motifs that are characteristic of nuclear receptor coactivators [10]. Second, studies have shown that forced expression of PPAR*γ*2 or PPAR*γ*1 can trigger the differentiation of fibroblasts to adipocytes resulting in the activation of adipocyte-specific genes and lipid accumulation [11].

The PPAR family consists of three isotypes that include PPAR*α* (NR1C1), PPAR*β* (also known as PPAR*δ*, NR1C2, FAAR, or NUC-1), and PPAR*γ* (NR1C3) [12–14]. A nuclear receptor, PPAR*γ*, is known to play a central role in fat metabolism and adipocyte differentiation [15, 16]. The PPAR*γ* is present in two key isoforms, PPAR*γ*1 and PPAR*γ*2. The two isoforms stem from alternate promoters [17]. Compared to PPAR*γ*1, PPAR*γ*2 has an additional 30 amino acids at the N-terminal end and is distinctively expressed in adipose tissue, where it plays a key role in adipogenesis [18]. These nonsteroidal receptors (i.e., do not mediate effects of steroids) form part of a class I nuclear hormone receptor superfamily [19] and function as ligand-activated transcription factors [20–22].

Each of the three PPAR isotypes is constitutively expressed in certain reproductive and nonreproductive rat tissues [23, 24], but their temporal and cell-specific expression in penile tissue, with the exception of a limited demonstration of PPAR*γ* in penile corporal smooth muscle cells [25], has not been shown. Further, no specific link is known between neonatal activation of ER*α* and penile PPAR*γ*. This is important given the expanded definition of the term endocrine disruptors to include activation of metabolic sensors such as PPARs. A number of findings suggest involvement of PPARs in endocrine disruption either through direct receptor activation or indirectly through crosstalk with other nuclear receptors. First, in vitro studies demonstrated that PPAR*γ* and ER*α* (the iconic receptor involved in endocrine disruption) are implicated in cross-talk [26–28]. Second, some endocrine disruptor chemicals, such as monethylhexyl phthalate (MEHP), a primary metabolite of diethylhexyl phthalate (DEHP), mediate their toxic effect by PPAR*γ* activation [29, 30]. Third, several nonbiological xenobiotics compounds can activate PPAR*γ*. For example, activation of PPAR*γ* with synthetic PPAR*γ* activators, such as antidiabetic drugs thiazolidinediones (TZDs), improve insulin sensitivity but they undesirably increase preadipocyte differentiation and white adipose tissue mass [31–33]. Consistent with this adipogenic effect, reduced PPAR*γ* level, as in mice with heterozygous (PPAR*γ* ^+/−^) deficiency, is associated with reduced white adipose tissue mass [34].

Findings related to interaction between ER*α* and PPAR*γ* in the aforementioned DES-penile rat model will illuminate a potential molecular mechanism by which estrogen exposure at critical period of development perturbs reproductive tissues. Therefore, we hypothesize that DES-induced penile adipogenesis is associated with ER*α*-mediated activation of PPAR*γ*. Objectives of this study were to (1) determine the basal expression of PPARs (*α*, *β*, and *γ*) in rat penis and (2) evaluate the neonatal modulatory effect of ER*α*-activator DES on penile PPAR*γ* as a marker of undesirable adipogenesis.

## 2. MATERIALS AND METHODS

### 2.1. Animals and treatments

This DES study was performed in collaboration with Dr. Hari Goyal at Tuskegee University using male pups from pregnant female Sprague-Dawley (SD) rats (Harlan Sprague-Dawley, Indianapolis, Ind, USA). All animal procedures were approved by Institutional Animal Care and Use Committee at Tuskegee University. In all experiments, rats were maintained using standard housing conditions including constant temperature of 22°C, *ad libitum* water and feeding, and 12:12 hours light dark cycle. Two experiments were conducted. In experiment 1, three groups of male pups (*n* = 5 per group, all were littermates) received subcutaneous injections of 25 *μ*L of olive oil (control), oil containing DES (0.1 mg/kg, Sigma-Aldrich, St. Louis, Miss, USA), or DES plus ICI 182, 780 (16.6 mg/kg, ICI; Tocris Bioscience, Ellisville, Miss, USA) daily on postnatal day 2 to 6. Rats in experiment 1 were sacrificed at 28 day of age. ICI 182, 780 is a high-affinity estrogen receptor antagonist (IC_50_= 0.29 nM) and is also considered a high-affinity ligand for the membrane estrogen receptor GPR30 (Tocris Bioscience). In experiment 2, two groups of male pups (*n* = 4 per group) received DES (1 mg/kg) or olive oil (control) every other day for 6 days starting at postnatal day 2. Penile tissues were collected from rats sacrificed at 120 days of age (adulthood). Small sections of the penile shaft tissue from each rat in experiment 1 and 2 were fixed overnight in 4% paraformaldehyde for IHC or fat staining, and the remainder of the shaft tissue was frozen in liquid nitrogen and stored at −80°C for RNA extraction and PCR analysis. The doses used for end-point evaluation at 28 and 120 days post-treatment were based on previous publications from our group that showed DES prenatal exposure (between postnatal days 2 to12) at a dose range of 0.1 to 0.12 mg/kg/day, or higher (1 mg/kg/day) result in similar abnormal penile development and adipogenesis [5, 8].

### 2.2. Total RNA isolation

Total RNA was isolated from the body of the penis using TRIZOL reagent (Invitrogen-Life Technologies Inc., Carlsbad, Calif, USA), according to the manufacturer's protocol. RNA concentrations were estimated at 260 nm and the ratio of 260/280 was determined using UV spectrophotometry (DU640, Beckman Coulter Fullerton, Calif, USA). The integrity of each RNA sample, indicated by the presence of intact 28S and 18S ribosomal RNA, was verified by denaturing agarose gel electrophoresis. RNA samples were treated with DNase (Ambion Inc.) to remove possible genomic DNA contamination. Samples with 260/280 ratio of ≥1.8 were used.

### 2.3. Conventional end-point and real-time PCR

Expression of mRNA for PPAR (*α*, *β*, and *γ*) isotypes was initially determined by conventional end-point RT-PCR with primers designed using primer quest software and synthesized by Integrated DNA Technology (IDT Inc, Coralville, Iowa, USA) from previously published rat sequences (see Table 1). Subsequently, semiquantitative RT-PCR for coamplification of PPARs and S-15 (known as Rig; small subunit ribosomal protein used as a house keeping gene) was performed to determine the relative expression levels of PPAR isotypes. Verification of accurate PCR products was confirmed by determination of the expected size of PCR bands and by sequence analysis of generated amplicons at Auburn University sequencing facility. The resulting sequences for the three PPAR isotypes were matched with previously published rat sequences in GenBank (accession number **NM013196**, **U40064**, and **NM013124** for PPAR*α*, PPAR*β*, and PPAR*γ*, resp.) using Chromas 2.31 software (Technelysium Pty ltd, Tewantin Qld 4565, Australia). PPAR*γ* splice variants or subtypes were identified using specific primers designed for rat PPAR*γ*1a and PPAR*γ*1b synthesized by IDT Inc. (Table 1). Liver and white adipose tissues from adult Sprague-Dawley rats in experiment 2 were used as positive controls for PPAR*γ*1 [35] and PPAR*γ*2 [18], respectively. The amplification protocol was as follows: initial cycle for 3 minutes at 95°C, and 30 cycles each at (95°C for 30 seconds, 55°C for 30 seconds, and 72°C for 30 seconds) followed by a final extension cycle at 72°C for 7 minutes. PCR reactions were performed on a Robocycler (Stratagene Inc, La Jolla, Calif, USA) and products were analyzed electrophoretically on 2% (w/v) agarose gels. The intensity of the PCR bands was determined using Fluor-S multi-imaging analysis system (Bio-Rad, Hercules, Calif, USA). Level of mRNA for PPARs was normalized to the levels of S-15 housekeeping gene.

Quantitative real-time PCR (Bio-Rad, MyiQ^TM^) for determination of expression levels of PPAR*γ* and ER*α* mRNA was performed in 25-*μ*L reaction mixture containing 12.5 *μ*L RT^2^ real-time SYBR/Fluorescein Green PCR master mix, 1 *μ*L first strand cDNA, 1 *μ*L RT^2^ validated PCR primer set for PPAR*γ* or ER*α* (Super Array Bioscience Corporation, Frederic, Md, USA), and 10.5 *μ*L PCR-grade water (Ambion Inc). Samples were run in 96-well PCR plates (Bio-Rad, Hercules, Calif, USA) in duplicates, and the results were normalized to GAPDH (see primer set in Table 1) expression. The amplification protocol was set at 95°C for 15 minutes for one cycle, and 40 cycles each at (95°C for 30 seconds, 55°C for 30 seconds, and 72°C for 30 seconds) followed by melting curve determination between 55°C and 95°C to ensure detection of a single PCR product. Template RNA from rat white adipose tissue and penis were used for determination of amplification efficiencies for (ER*α*/PPAR*γ*) targets and GAPDH by generating standard curves. Curves were generated by using serial 10-fold dilutions total RNA and plotting the log dilution against C_T_ (threshold cycle) value obtained for each dilution. The Pearson's correlation coefficient (*r*) value for each generated standard curve was ≥0.98, and the calculated amplification efficiency was between 98.5 to 99%.

### 2.4. Immunohistochemistry (IHC)

Immunolocalization of PPAR*γ* in penile tissue was performed using mouse anti-PPAR*γ* IgG1 monoclonal antibody (sc7273, Santa Cruz Biotechnology Inc, Santa Cruz, Calif, USA) raised against a C-terminus sequence of human and mouse PPAR*γ* (similar to the corresponding rat sequence). The antibody detects PPAR*γ*1, PPAR*γ*2 and, to a lesser extent, PPAR*α* and PPAR*β* of rat, mouse, and human by IHC using paraplast-embedded tissues. Approximately 5-mm-long penis sections from the middle of the body of the penis were fixed in 4% paraformaldehyde for 48 hours, embedded in Paraplast (Sigma-Aldrich), and cut at 5-*μ*m thickness [7]. Mounted penis sections were deparaffinized in Hemo-D (Scientific Safety Solvents, Keller, Tex, USA) and hydrated to distilled water (dH_2_O). The slides were transferred to a rack and placed in 1 L of 10 mM sodium citrate (pH 6.0). The beaker was placed on a hot plate, allowed to come to a boil and tissues were boiled for 20 minutes. When the citrate solution cooled to near room temperature (RT), the slides were transferred to a glass staining dish and equilibrated in phosphate buffered saline (PBS) (Sigma-Aldrich, ST Louis, Miss, USA). After 20 minutes incubation in blocker (5% normal goat serum, Sigma-Aldrich) and 2.5% BSA (Sigma) in PBS, slides were washed briefly in PBS. Anti-PPAR*γ*, diluted 1:20 in blocker, was applied and the sections were left to incubate overnight at RT. Next day, slides were washed 3x in PBS, 3 minutes each, and tissues were incubated with Alexa 488-conjugated goat antimouse IgG (Molecular Probes, Eugene, Ore, USA) for 1 hour at RT. After washing two times in PBS, 3 minutes each, slides were mounted with VectaShield (Vector Laboratories, Burlingame, Calif, USA), and the coverslips were sealed. The sections were examined using a Nikon TE2000E microscope and digital images were generated using an attached Retiga EX CCD digital camera (Q Imaging, Burnaby, BC, Canada). Penile tissue sections from all 28-day treated rats were examined. Representative micrographs from different penile histological structures were shown for untreated control rats, and for rats treated with DES or DES + ICI.

### 2.5. Fat staining

Histochemical demonstration of fat was performed as previously described [7]. Briefly, tissue sections from penile body, approximately 5 mm-long, were fixed for 24 hours in 4% formaldehyde, followed by en bloc staining of fat for 8 hours with 1% osmium tetroxide dissolved in 2.5% potassium dichromate solution. Specimens were then processed for paraplast embedding and cut at 5-*μ*m thickness. Deparaffinized sections were examined for black staining indicative of fat cells using light microscopy.

### 2.6. Statistical analyses

Analysis of real-time PCR data for relative gene expression level (fold change of target relative to control) was performed using a modification of the delta delta Ct method (ΔΔ CT) as described previously [36]. Statistical differences between treatment groups were performed using Sigma Stat statistical software (Jandel Scientific, Chicago, Ill, USA). Δ CT for real-time PCR data [37], and intensity values (for semi-quantitative RT-PCR data) were subjected to analyses of variance. Experimental groups with means significantly different (*P* < .05) from controls were identified using Holm-Sidak and Tukey tests. When data were not distributed normally, or heterogeneity of variance was identified, analyses were performed on transformed or ranked data.

## 3. RESULTS

### 3.1. Detection and sequence analysis of PPAR and ER*α* transcripts in the body of the penis

**Figure 1:**
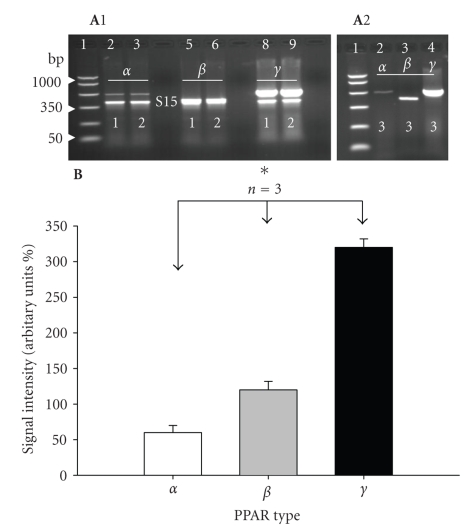
(A1) and (A2) RT-PCR amplification of three PPARs (*α*, *β*, and *γ*) from the body of the penis of three (1, 2, and 3) normal adult (120 days) control rats. (A1) Shows coamplification of PPARs (*α*, *β*, and *γ* (upper bands) and S15 (small ribosomal subunit protein as housekeeping gene, lower bands) in two representative rats (1 and 2). PCR markers were included in lane 1. Expected band sizes for S-15, PPAR*α*, PPAR*β*, and PPAR*γ* were 361, 492, 390, and 533 bp, respectively. Identities of amplicons were further confirmed by sequence analysis (see Section 2). Note that the ampilicons for PPAR*β* and S15 in lane 5 and 6 were overlapped (compare run for PPAR*α*, PPAR*β*, and PPAR*γ* without S15 shown for rat 3 in (A2). In all rats note the predominant expression of PPAR*γ*. 15 *μ*L PCR products were loaded per each lane. (B) Graphic representation of signal intensity for PPARs showing predominant expression of PPAR*γ*. Transcript levels were normalized to the levels of S15 housekeeping gene. To calculate the intensity for PPAR*β* the mean intensity of S-15 in lanes 2, 3, 8, and 9 in Figure (A1) was subtracted from the combined intensity of PPAR*β* + S15 in lanes 5 and 6 to obtain the intensity of penile PPAR*β* for rat 1 and 2, respectively. *P < .05.

**Figure 2:**
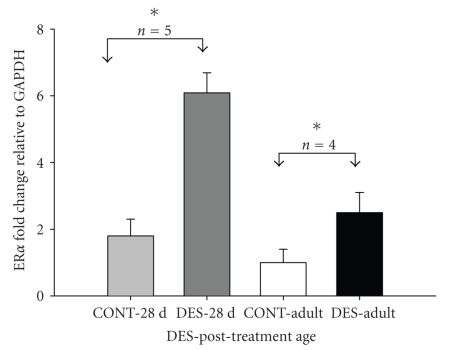
Real-time PCR showing 3.38 and 2.5 fold increase in ER*α* mRNA in penile tissue of 28-day-old (DES-28 d) and adult rats (DES-Adult) neonatally treated with DES, respectively. Fold change was calculated relative to respective controls (CONT-28 d and CONT-Adult). Data (*n* = 4-5) are expressed as mean ±SE. *P < .05.

**Figure 3:**
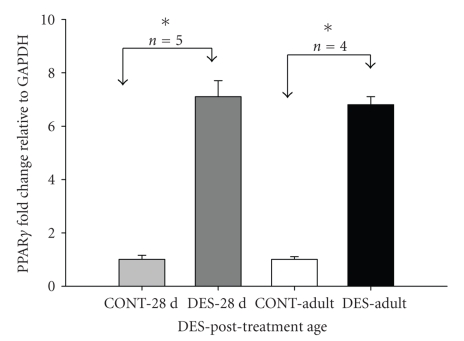
Real-time PCR showing 7.1 and 6.8 fold increase in PPAR*γ* mRNA in penile tissue of 28-day-old (DES-28 d) and adult rats (DES-Adult) neonatally treated with DES, respectively. Fold change was calculated relative to respective controls (CONT-28 d and CONT-Adult). Data (*n* = 4-5) are expressed as mean ±SE. *P < .01.

**Figure 4:**
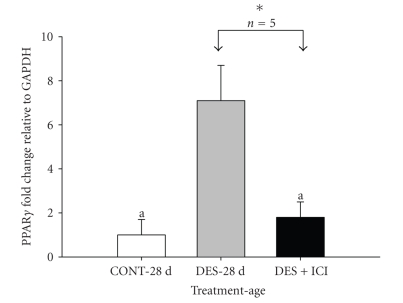
Real-time PCR data showing attenuation of the effect of DES on PPAR*γ* mRNA by ER blocker ICI 182, 780 in 28-day-old rats treated neonatally with either 25 *μ*L of olive oil (CONT-28 d), oil containing DES (DES-28 d; 0.1 mg/kg bw), or DES plus ICI (DES + ICI; 16.6 mg/kg). ICI treatment significantly inhibited DES-induced PPAR*γ* mRNA [DES-28 d versus DES + ICI]. Comparison between control and DES treated rats showed 7.1 fold increase in expression [CONT-28 d versus DES-28 d]. Letter [a] indicates no significant differences between CONT-28 d and DES + ICI. Data (*n* = 5) are expressed as mean ±SE. *P < .05.

Primer sets used in this study are shown in Table 1. Transcripts for three PPAR isoforms (*α*, *β*, and *γ*) were detected, albeit at different levels, in penile tissue from normal control adult (120 days) rats (Figure 1, parts A1 and A2). Semiquantitative RT-PCR analysis of PPARs indicated predominant expression of PPAR*γ* mRNA when compared with PPAR (*α* and *β*) isoforms (Figure 1(B)). Sequence analysis and alignment with published sequence data confirmed the identity of all three PPAR isoforms. Treatment with DES induced over three-fold-increase (3.38) in ER*α* transcripts in 28-day-old rats compared to over two-fold-increase (2.5) in 120-day-old adult rats when each age group was compared with its respective untreated controls (Figure 2). Similarly, DES induced slightly over seven-fold-increase (7.1) in PPAR*γ* transcription level in 28-day-old rats compared with over six-fold-increase (6.8) in 120-day-old adult rats (Figure 3). The upregulation of PPAR*γ* expression by DES in 28-day-old rats was abrogated when rats were cotreated with DES and ICI 182, 780 (Figure 4). The differences in the transcriptional level of penile ER*α* and PPAR*γ* between the DES-treated rat groups (28 versus 120-day-old rats) were not significantly different. Because of the relatively high expression of penile PPAR*γ* in the 28-day-old rats subsequent studies for determination of splice variants and PPAR*γ* protein expression were performed in the 28-day-old rats.

### 3.2. Detection of PPAR*γ* splice variants and real-time PCR data

**Figure 5:**
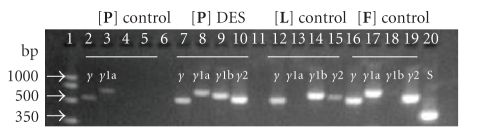
RT-PCR amplification of PPAR*γ* (*γ*, *γ*1a, *γ*1b, and *γ*2) splice variants in the body of the penis (**P**) of control (lanes, 2–5) and DES-treated (lanes, 7–10) 28-day-old rats. Lanes 12–15 were amplification products from RNA template obtained from rat liver (**L**) (used as positive control for PPAR*γ*1b). Lanes 16–19 were RNA template from rat white adipose tissue (**F**) (used as positive control for PPAR*γ*1a and PPAR*γ*2). Note that only PPAR*γ* and PPAR*γ*1a were detected in (**P**) of normal rats. In contrast, in DES-treated rats enhanced expression of all PPAR*γ* splice variants can be noted. In addition to PPAR*γ* and PPAR*γ*1a expression (seen in normal rats), PPAR*γ*1b and PPAR*γ*2 were induced by DES-treatment. As expected, PPAR*γ*1b and PPAR*γ*2 were strongly expressed in (**L**) and (**F**), respectively. S-15 (S, lane 20) is a housekeeping gene amplified from (**P**) as control for RT-PCR conditions. The expected amplicon sizes for S, PPAR*γ*1a, PPAR*γ*1b, PPAR*γ*2, and PPAR*γ* are 361, 658, 618, 563, and 533 bp, respectively.

In order to determine which PPAR*γ* splice variant is expressed in the body of normal and DES-treated rats, primers (Table 1) were designed to amplify the two known rat PPAR*γ*1a and PPAR*γ*1b splice variants using conventional end-point RT-PCR. Splice variant analyses revealed expression of PPAR*γ*1a in normal 28-day-old rat penis. However, in addition to PPAR*γ*1a, PPAR*γ*1b and PPAR*γ*2 were newly induced by DES treatment (Figure 5).

### 3.3. Immunohistochemistry and fat staining

**Figure 6:**
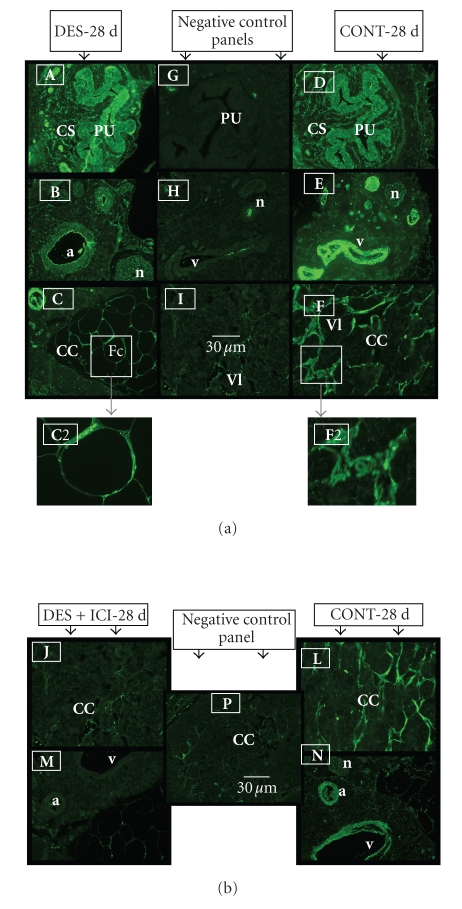
(a) Representative immunohistochemical staining for PPAR*γ* protein in the body of the penis of 28-day-old DES-treated (A), (B), and (C) and control untreated rats (D), (E), and (F). Note that PPAR*γ* protein is expressed in DES-treated (DES-28 d) and normal rats (CONT-28 d) with increased intensity and fat cells in DES-treated rats (see panel (C)). Note expression in transitional epithelium of the penile urethra (PU) and the surrounding corpus spongiousm (CS) in (A) and (D) and in the endothelium of blood vessels and smooth muscle cells in the dorsal artery (a) and vein (v), and in nerve fibers of the dorsal nerve (n) of the penis (B) and (E). Similar staining intensity can be seen in the endothelium and smooth muscles of the vascular lacunae (Vl) in the corpus cavernosum penis (CC) in control normal rats (F). Note one contrasting difference is that the cavernous spaces in DES-treated rats in panel (C) are replaced with fat cells (Fc) that show increased staining intensity in the cell nucleus located at cell periphery. Panels (C2) and (F2) show a closer view of area outlined by insert box. Control sections (minus primary antibody) were in panels (G), (H), and (I). Scale bar = 30 *μ*m. (b) IHC staining for PPAR*γ* protein was significantly reduced by ICI 182, 780 treatment [compare staining in panels (J) and (M) with (L) and (N)]. Panel (P) is a negative control (minus primary antibody). Scale bar 30 *μ*m.

**Figure 7:**
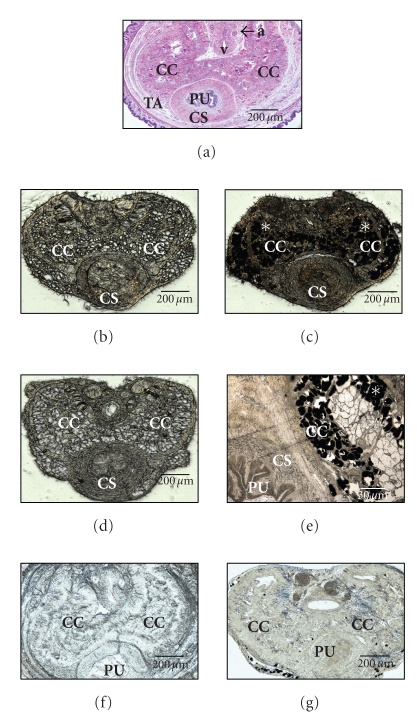
Micrograph sections from penile body of normal rat (a) and rats treated neonatally with DES (b)–(e) or DES + ICI (f)-(g). Panel (a) was from a normal adult rat stained with H and E for demonstration of normal histological structures of the penis (a: dorsal artery; v: dorsal vein; CC: corpus cavernosum; CS: corpus spongiosum; PU: penile urethra; TA: Tunica albuginea). Panels ((b), unstained) and ((c), stained for fat with 1% osmium tetroxide) were from a 28-day-old rat. Panels ((d), unstained) and ((e), stained for fat with 1% osmium tetroxide and presented as a magnified view of CC and CS regions) were from adult rat (120 days) treated neonatally with DES. Note the empty appearing spaces of fat cells in CC regions in unstained sections (panels (b) and (d)). In sections stained with 1% osmium tetroxide (to confirm presence of fat) fat cells appear as black granules, *. Panels ((f), unstained) and ((g), stained with 1% osmium tetroxide) were from a 28-day-old rat treated neonatally with DES + ICI. Note the absence of empty appearing fat cells and lack of fat staining in CC region. Sections from these rats were used for immunolocalization of PPAR*γ* in Figure 6 parts (a) & (b). Scale bars = 30 (E) and 200 *μ*m in other panels.

Immunohistochemistry results revealed PPAR*γ* protein localization in transitional epithelium of the urethra, and the surrounding corpus spongiosum penis. It is also expressed in stromal, endothelial, neuronal, and smooth muscular cells of the cavernous sinuses located in the corpus cavernousm region of normal 28-day-old rat penis (Figures 6(a) and 6(b)). Treatment with DES induced a strong staining intensity for PPAR*γ* protein in the peripherally located nuclei of newly induced adipocytes (Figure 6(a), Panel (c) with a magnified inset-box view in C2). PPAR*γ* immunostaining was markedly reduced by ICI 182,780 treatment (Figure 6(b)). In unstained penile sections from 28-day-old and adult DES-treated rats, the new adipocytes were seen as empty spaces similar to fat cells and were specifically localized in the corpus cavernosum region of the penis (Figure 7, panels (b) and (d)). In addition, staining with 1% osmium tetroxide confirmed that the empty spaces were cluster of fat cells (stained as black granules in Figure 7, panels (c) and (e)). No fat cells were seen in penile sections from rats treated with DES + ICI (Figure 7, panels (f) and (g)).

## 4. DISCUSSION

This study demonstrated that three PPAR transcripts (*α*, *β*, and *γ*) are constitutively coexpressed in normal rat penis with PPAR*γ* as the predominant isotype. In addition, it established that some ER*α* synthetic ligands, such as DES, can activate PPAR*γ* subtypes when administered at early perinatal days. Further, upregulation of ER*α* by DES was associated with a corresponding increase in PPAR*γ* suggesting a synergistic interaction between the two receptors.

Previous studies that used in situ hybridization to determine the distribution of PPARs in rat tissues, including reproductive organs, showed expression of PPAR*α* and PPAR*β* in somatic (Sertoli and Leydig) and in germ cells of the testis, but did not address expression of these two receptors in penile tissue [23, 24]. The role of PPAR*α* and PPAR*β* in the testis, however, remains unknown. Detailed study addressing expression of PPAR*γ* isotypes in penile tissue is also lacking, with the exception of a study that showed limited penile PPAR*γ* expression in corporal smooth muscle cells [25].

In this study, PPAR*γ* and PPAR*γ*1a were detected in normal rat penis. However, DES as ER*α* activator distinctively induced expression of PPAR*γ*1b and PPAR*γ*2 splice variants that were not present in control untreated penile tissue. The induction of splice variant PPAR*γ*1b is in agreement with previous in vitro studies that demonstrated activation of PPAR*γ*1 by the endocrine disruptor monoethyl-hexyl-phthalate in C2C12 mouse skeletal muscle cell line [2], and with MCF-7 breast cancer cells stimulated with E2, the natural ER*α* ligand [38]. Further, the induction of PPAR*γ*2 concurs with increased adipogenesis observed in the corpus cavernousm penis as PPAR*γ*2 is considered a unique marker for mature adipocytes, and its forced induction is associated with terminal differentiation of preadipocytes or fibroblast cells to functional mature adipocytes [11, 22]. The upregulation of PPAR*γ* was abrogated by coadministration of the type-II antiestrogen ICI 182,780, indicating that DES effects were mediated, at least in part, via the estrogen receptor system. It is possible, however, that ICI may have directly repressed activation of PPAR*γ* as ICI was previously shown to inhibit the action of the selective PPAR*γ* agonist BRL 48, 482 in MDA-MB 231 breast cancer cell culture in the absence of ER [38].

One important difference between this study and previous in vitro studies that addressed signal cross-talk between PPAR*γ* and ER*α* using MCF-7 cells [38–40] is that the activation of ER*α* by DES in our study is associated with selective induction of PPAR*γ*1b and PPAR*γ*2. This unique effect resulted in generation of de novo adipocytes that provide direct functional proof for PPAR*γ*2 induction. In contrast to our study, activated ER*α* by E2 lowers both basal and ligand-stimulated PPAR*γ*-mediated gene reporter activity in MCF-7 cancer cell culture [38]. Likewise, activation of PPAR*γ* in MCF-7 cell culture with the natural PPAR*γ* ligand cyclopentenone 15-deoxy-Δ^12,14^ prostaglandin J2 (15d-PGJ2) inhibited estrogen-responsive elements [40]. Consequently, the MCF-7 cell culture studies suggest that ER*α* and PPAR*γ* negatively regulate each other. The reason for the difference between our study and the aforementioned in vitro data could be related to differences between in vitro and in vivo milieu or to the deletional mutants used in the in vitro studies compared with the in vivo wild type receptors in our study. Another reason for the disagreement could be due to differences in coactivators and corepressors present in MCF-7 and penile tissue cells or more importantly to differences in the ligands used. One plausible hypothesis, however, for the increased transcriptional activation of PPAR*γ*1b and PPAR*γ*2 by DES-activated ER*α* is that exposure of rats to DES at a critical neonatal period of days 1 to 12 is uniquely associated with reprogramming of penile stromal or preadipocytes to mature adipocytes [5–8]. In support of this concept, it is known that postnatal days 1 to 5 in rodents coincide with a period for reproductive tract and adipocyte differentiation [41]. Further, data from other laboratories indicated that neonatal exposure of rodents to DES is associated with increased whole body fat at adulthood [42]. This novel adipogenic effect of DES was proposed as a model for the study of what is called developmental obesity mediated by early exposure to endocrine disruptors [43].

The molecular mechanism involved in DES-ER*α*-PPAR*γ* transactivation could be related to two factors. First, activated-ER*α* could directly bind to PPAR response elements (PPREs) because the two receptors share the capacity to bind to the AGGTCA half-sites consensus sequences contained as palindrome or direct repeat in estrogen response elements (EREs) and PPRE sequences, respectively [44]. This mechanism could result in bidirectional activation of shared target sequences between ER*α* and PPAR*γ* depending on activated receptor involved. Second, it is known that estrogen could induce enzymatic conversion of prostaglandin D2 (PGD2) and the endogenous metabolites of the latter can directly activate PPAR*γ* [45]. The latter effect, however, was not associated with induced PPAR*γ* mRNA [46] suggesting that the first mechanism could be in play in our study.

The strong PPAR*γ* protein expression in normal transitional epithelium of the urethra and the dorsal artery and vein of the penis indicates possible physiological role for PPAR*γ* in the penis vasculature and the urothelium of the urinary tract. Although this study did not address functionality of PPAR*γ* in the penis, current evidence suggests that its constitutive expression in some tissues is linked to eicosanoids and prostaglandins (PGs) actions [47, 48]. In this regard, the terminal metabolite of the J series of PG, 15d-PGJ2, is considered the natural activator of PPAR*γ* [48]. Sources of penile PGs could include synthesis by local penile cells and/or cells of the renal medulla where PGs can be transported via the ureter and pelvic urethra to the penis [49]. Among other functions, PGs are important mediators of inflammation, vascular homoeostasis, and pain all of which may be relevant to the pathophysiology of the penis.

Staining with osmium confirmed the presence of new lipid-laden adipocytes in penile tissues of DES-treated rats. Previously, our group showed that Sprague-Dawley rats treated neonatally with DES accumulated fat in the corpus cavernous penis [5–8] just as observed for the rats in the present study. The histological demonstration of DES-induced lipid buildup in the corpus cavernosum penis concurs with the newly induced adipocyte marker PPAR*γ*2 detected with RT-PCR.

In penile tissue direct pharmacological activation of PPAR*γ* by the antidiabetic TZD pioglitazone reportedly blocked corporal veno-occlusive dysfunction in rat model of type 2 diabetes mellitus [25]. However, this effect was associated with fat buildup suggesting that direct activation of penile PPAR*γ* by TZDs or indirectly by ER*α* ligands, as in this study, could be a potential pathway for development of undesirable adipogenesis. In conclusion, PPARs are currently considered potential drug targets for diverse conditions including, vascular homoeostasis, diabetes mellitus, hyperlipidemia, inflammation, cancer, and infertility [50–54]. This study furthers our knowledge of mechanisms of endocrine disruption mediated by PPAR*γ* in male subjects. The ER*α*-PPAR*γ* signal pathway activation by DES is analogous in some way to mechanisms postulated for endocrine disruptor MEHP and other phthalates esters and organotins which directly activates PPAR*γ* and promotes adipogenesis in cell culture models [2, 3, 55].

## Figures and Tables

**Table 1:** PCR primer sets, sequence, product size (bp), nucleotide (nt) location, and GenBank accession numbers for rat PPARs used in this study. Note that a common antisense oligoprimer (sequence in bold) was used for PPAR*γ*1a, PPAR*γ*1b, and PPAR*γ*2.

Product/	Sense primer	Antisense primer	Product size	nt
accession#			(bp)	location
PPAR*α*	5-TTG TGA CTG GTC AAG CTC AGG ACA-3	5-TCG TAC GCC AGC TTT AGC CGA ATA-3	492	296–787
**NM013196**				

PPAR*β*	5-TAA CGC ACC CTT CAT CAT CCA CGA-3	5-TTG ACA GCA AAC TCG AAC TTG GGC-3	390	873–1262
**U40064**				

PPAR*γ*	5-TCT CCA GCA TTT CTG CTC CAC ACT-3	5-ATA CAA ATG CTT TGC CAG GGC TCG-3	533	257–789
**NM013124**				

PPAR*γ*1a	5-CTG ACG AGG TCT CTC TC G GCT G-3	**5-AGC AAG GCA CTT CT GAA ACC GA-3**	658	21–679
**AF246458**				

PPAR*γ*1b	5-CAG CGC TAA ATT CAT CTT AAC T-3	**5-AGC AAG GCA CTT CTG AA A CCG A-3**	618	21–639
**AF246457**				

PPAR*γ*2	5-GAG CAT GGT GCC TTC GCT GA-3	**5-AGC AAG GCA CTT CTG AA A CCG A-3**	563	37–600
**AB019561**				85–648
**AF156666**				86–649
**Y12882**				

PPAR*γ*/ER*α*	(primers for real-time PCR were obtained from Superarray Inc)		190/179	[PPAR*γ*/ER*α*]
**NM013124** [PPAR*γ*]	(Sequence are not disclosed by the Company)			respectively
**NM012689** [ER*α*]				

Gapdh	5-ATG ATT CTA CCC ACG GCA AG-3	5-CTG GAA GAT GGT GAT G CGT T-3	89	71–159
**DQ403053**				184–272
**BC087743**				216–304
**BC059110**				

Rig/S15 (Ambion)	5-TTC CGC AAG TTC ACC TAC C-3	5-CGG GGC CGG CCA TGC T TTA CG-3	361	74–433
**BC105810**				
